# Psychometric Evaluation of the School Climate and School Identification Measure—Student on Chilean Students: A Bifactor Model Approach

**DOI:** 10.3390/children11010087

**Published:** 2024-01-11

**Authors:** José Luis Gálvez-Nieto, Ítalo Trizano-Hermosilla, Karina Polanco-Levicán

**Affiliations:** 1 Departamento de Trabajo Social, Universidad de La Frontera, Temuco 4780000, Chile; jose.galvez@ufrontera.cl; 2 Departamento de Psicología, Universidad de La Frontera, Temuco 4780000, Chile; 3 Programa de Doctorado en Ciencias Sociales, Universidad de La Frontera, Temuco 4780000, Chile; k.polanco01@ufromail.cl; 4 Departamento de Psicología, Universidad Católica de Temuco, Temuco 4780000, Chile

**Keywords:** school climate, adolescents, school, psychometric properties, bifactor

## Abstract

School climate is a relevant construct for understanding social relations at school. The SCASIM-St has been widely defined as a multidimensional construct; however, new factor structures have not been explored through evidence that allows for interpreting school climate scores from an approach that respects the multidimensionality of the scale and, at the same time, allows for identifying the degree of essential unidimensionality in the data. Consequently, the objective was to analyze the psychometric properties of the SCASIM-St from a bifactor model approach, evaluating the influence of a general school climate factor versus five specific factors. The study involved 1860 students of both sexes (42% males and 58% females), with an average age of 16.63 years (SD = 0.664), from 17 secondary schools in Chile. The results obtained by a confirmatory factor analysis provided evidence that the best model was the bifactor model for the 38 items, with one general factor and five specific factors. The Explained Common Variance (ECV) values and reliability levels by hierarchical omega accounted for a strong general school climate factor with high levels of reliability. Evidence of external criterion validity, assessed through the attitude toward authority scale (AIA-A), showed a theoretically expected and significant relationship between the factors of both instruments. This study confirmed the psychometric robustness of the SCASIM-St scale by means of a bifactor model, allowing for a new, essentially unidimensional interpretation of the scale scores and providing an instrument to measure school climate in Chile.

## 1. Introduction

School climate has implications at the academic, personal and relational levels that favor greater student adjustment [1,2,3]; thus, school climate is considered a relevant factor in achieving better academic performance [4,5,6] and greater school satisfaction [7]. The above is fundamental considering that adolescents are in a process of constant development and learning from their daily interactions; therefore, the way in which they relate among students and with teachers, as well as problem solving, influences their beliefs and their present and future behaviors [8,9].

From a conceptual perspective, school climate is understood as the quality of social relationships among the people who are part of a school community, such as students, teachers and professional staff, considering the academic and moral values aspects and the norms shared in the school context [10]. Consequently, an adequate school climate is associated with gratitude, prosocial behavior [11,12], perceived social support and the ability to overcome obstacles faced in daily life to achieve proposed objectives [13]. Along the same lines, school climate is positively related to adolescents’ psychological adjustment [2] and negatively related to externalizing and internalizing behaviors [2,12,14]. In addition, it moderates the association between homophobic victimization and depression [15], decreasing behavioral problems and peer conflicts and favoring school safety [16]. Consequently, a good school climate reduces school-related stressors [17], making students feel comfortable exposing themselves to different daily activities, thus improving their school experiences [18].

School climate is positively related to academic success [4,5,19,20,21], academic achievement and student motivation [21]. It is highlighted that positive perceptions of school climate were significantly associated with high levels of socionormative expectations, that is, with students’ high expectations about the future academic performance of their peers, which occurs in diverse socioeconomic environments [22]. In addition, an adequate school climate promotes learning for all students, especially those who may present some difficulty, such as hyperactivity/inattention [16]. Another relevant element is a structure that, together with the support for students, favors better student behavior when learning assessments are conducted [23].

With respect to the relationship between teachers and students, it can be noted that affectionate and respectful interactions favor opportunities for students to make decisions about their own learning and about those activities they would like to carry out, promoting their participation [17]. In addition, organization and respect for teachers reduce risk behaviors such as cannabis and alcohol consumption [24], and teacher support for students is negatively associated with an addiction to Internet games [25] and hostility, encouraging students to seek help, which would help to care for their mental health [2]. However, there is evidence that positive feedback, i.e., teachers’ approval of students’ behavior and their performance of academic activities, has a positive influence on students’ attitude, behavior in the classroom and their performance on their homework [26,27,28].

On the other hand, an adequate school climate is associated with a positive attitude toward authority; this association is mediated by students’ values [29,30]. Specifically, Del Moral et al. [31] report that adolescents who manifest aggressive behaviors toward their parents, who are authority figures, also show a less positive attitude toward institutional authority. This shows a major attitude toward the transgression of the rules that govern the behavior of students in order to achieve adequate coexistence in their schools and a perception of school climate as deficient considering student class participation, their relationships with other students and the attention and help provided by the teacher toward their students. Thus, it is observed that adolescents’ violent behaviors are directly and negatively related to their positive attitude toward authority and directly and positively related to their attitude toward the transgression of social norms [32,33,34], affecting other students who are subject to aggressive behaviors in a physical and relational way [35].

Lee et al. [10] developed and proposed the dual School Climate and School Identification Measure—Student (SCASIM-St) scale. This scale is based on Bronfenbrenner’s [36] ecological systems theory, which considers that students’ behavior reflects the influence of the various social contexts in which they participate [37]. The SCASIM-St scale presents a second-order multidimensional factor structure called school climate that is composed of four first-order factors: Student–Student Relations, which refer to the social relationships among peers; Student–Staff Relations, which allude to the relationship established between students and school administrative staff; Academic Emphasis, which focuses on the degree of support a student receives from a teacher to achieve learning goals; and finally, Shared Values and Approaches, which assess whether a student shares a school’s goals and norms. In addition, a fifth first-order factor related to the second-order factor, called School Identification, assesses whether belonging to their school is part of a student’s identity.

According to Lee et al.’s [10] proposal, the SCASIM-St articulates two theoretical concepts: school climate and school identity. According to this theoretical proposal, school climate and school identification favor a student’s feeling of belonging with respect to their educational community and their school. In this way, it allows for evaluating students’ construction of identity, considering the social groups they are integrated within and the cognitive and emotional valuation they give them, since these influence their beliefs and individual behaviors [38,39,40]. In this sense, the perception of belonging gradually and progressively influences the internalization of the norms and values of the reference group, influencing what they evaluate as adequate or acceptable in their social environment [10,38,39,40]. Along the same lines, school identification and school climate are related to academic achievement [41,42], noting that academic achievement in writing and mathematics is associated with school climate through students’ identification with their school [43]. However, research results in Chilean adolescents show that school identification is negatively related to a positive attitude toward the transgression of school rules by students and positively related to a positive attitude of adolescents toward authority figures, which, in this case, are teachers and other adults who perform different tasks in the educational community [44,45].

The SCASIM-St has been adapted and validated in different languages and countries. This instrument has two versions: a long version and an abbreviated version. The long version is composed of 38 items following the previously mentioned second-order structure. For the long version, the first study was applied first to a sample of Australian students [10], then a second psychometric study was conducted on Turkish students [46] and a third study was carried out on a sample of Chilean adolescents [47]. Regarding the abbreviated version of the SCASIM-St15, this version presents a 15-item structure, divided into five first-order factors and one second-order factor. The short version of the scale has been studied twice. It was first applied to a sample of Chilean adolescents [45] and, in a more recent study, to Chinese adolescents [48]. Both the long version and the abbreviated version of the SCASIM-St have shown a stable factorial structure, presenting psychometric indicators that support the original theoretical structure.

Based on the above, it is evident that focusing on school climate in schools is fundamental for all members of the educational community [14,16,17]. In this sense, visualizing and understanding the importance of the role of school climate is crucial to achieving an environment that enhances the proper development of students [49]. Likewise, the need to improve the quality of school climate assessments, distinguishing between the global aspects and specific factors of this construct, can improve intervention approaches, facilitating decision making. Considering the empirical evidence that demonstrates the relevance of school climate and the possibility of expanding new interpretations regarding the factorial structure, this study aimed to analyze the psychometric properties of the SCASIM-St from a bifactor model approach, evaluating the influence of a general school climate factor versus five specific factors for a sample of Chilean adolescents.

Consequently, this study will test the following hypotheses:

**H1.**  *The SCASIM-St scores will present a factor structure congruent with the theoretical proposal of a general second-order school climate factor;*

**H2.**  *The SCASIM-St scores will present a bifactor structure, with the predominance of a general school climate factor and five specific factors, presenting a better fit than the second-order model;*

**H3.**  *The scores will present adequate evidence of reliability for their use in the Chilean context;*

**H4.**  *The school climate will be positively related to a positive attitude to authority and negatively related to a positive attitude to transgression.*

## 2. Materials and Methods

### 2.1. Research Design

In order to answer the research objectives, this study applied a cross-sectional design, where the psychometric properties of the SCASIM-St scale were analyzed [50]. (see Supplementary Materials).

### 2.2. Participants

The population of this study totaled 22,469 students from municipal (6991), subsidized (14,224) and private (1254) schools. The selection of participants was made by means of stratified probability sampling, with a confidence of 99.7%, a variance of *p* = q = 0.50 and a margin of error of 3.8% [51].

The sample consisted of 1860 students from Chile of both sexes (42% male and 58% female). The age range was between 13 and 20 years, with a mean age of 16.63 years (SD = 0.664). The students came from 17 high schools: technical–professional (vocational) (61.8%) and scientific–humanistic (38.2%).

### 2.3. Instruments

A sociodemographic questionnaire was applied, which collected information related to age, sex and grade (school level).

In addition, the adapted version of the Dual School Climate and School Identification Measure—Student (SCASIM-St) [47] was applied. The SCASIM-St is a dual measure that assesses school climate and school identification [10]. It is a self-report scale that measures both constructs from 38 items that are responded to from a five-point ordinal scale (1 = strongly disagree; 5 = strongly agree). This scale has a factor structure of four first-order factors: Student–Student Relations (seven items, e.g., “Students are friendly to each other”), Student–Staff Relations (nine items, e.g., “Staff care about students”), Academic Emphasis (eight items, e.g., “Teachers challenge students to do better”) and Shared Values and Approaches (eight items, e.g., “The school values and goals are well understood”). These four factors are grouped into a second-order factor called school climate. The SCASIM-St also presents a fifth factor related to the second-order factor called School Identification (six items, e.g., “I feel a strong connection with this school”) [10]. Reliability and validity evidence demonstrated adequate psychometric properties for use in Australia [10], Turkey [46] and Chile [47]. In the Chilean context, it was validated by a linguistic adaptation procedure [47], presenting favorable reliability indicators with values ranging between a maximum McDonald Omega score = 0.916 (Student–Staff Relations) and minimum McDonald Omega score = 0.840 (Student–Student Relations). The validity indicators showed satisfactory values: WLSMVχ^2^ (660) = 4553.020, *p* < 0.001; CFI = 0.958; TLI = 0.956; RMSEA = 0.055 (CI90% = 0.053–0.056).

Finally, the adapted version of the Attitudes to Institutional Authority in Adolescence Scale (AIA-A) [44] was applied. The AIA-A is a self-report scale that assesses adolescents’ attitudes toward authority figures. It has 9 items and is answered using a five-point ordinal scale (1 = never; 5 = always). The factor structure of the AIA-A is composed of two factors: Positive Attitude to Authority (5 items, e. g., “The police are there to make society better for everyone”), referring to the degree of respect toward teachers and the police, and Positive Attitude to Transgression (4 items, e. g., “It is normal to break the law if you do not cause harm to anyone”), referring to positive attitudes toward the transgression of school rules. Reliability and validity evidence demonstrated adequate psychometric properties for its use in Spain and Mexico [52] and Chile and Colombia [44]. Regarding the adaptation of this scale for Chile, the AIA-A was adapted by a linguistic adaptation procedure [41]. This instrument showed satisfactory reliability indicators, both for the Positive Attitude to Authority factor (Cronbach’s alpha = 0.712) and for the Positive Attitude to Transgression factor (Cronbach’s alpha = 0.756). The construct validity indicators showed satisfactory values: SB-χ^2^ (26) = 119.425, *p* < 0.001; CFI = 0.929; TLI = 0.902; RMSEA = 0.072 (IC90% = 0.059–0.085).

### 2.4. Procedure

Communication was established with the school principals to manage the application of the scales, formally requesting them to sign a collaboration agreement with the research team. Subsequently, informed consents were sent to the students’ parents, and once the corresponding authorizations were obtained, an informed consent form was applied to the study participants. After guaranteeing the integrity of the ethical principles of the project, the measurement instruments were managed using the QuestionPro platform and were applied by the technical personnel assigned to the project during the first hour of classes.

Participants were selected in a multistage manner. First, educational establishments were randomly selected, considering the following stratification variables: geographic area, type of education and type of establishment (public or private). Second, two classes per educational establishment were randomly selected. The percentage of missing data did not exceed five percent of the total sample.

### 2.5. Data Analysis

Initially, descriptive analyses of the center, dispersion and shape statistics were carried out for each of the 38 items of the SCASIM-St. The MPLUS v.8.1 software [53] was used to evaluate the latent structure of the instrument. Due to the ordinal nature of the items and the absence of normality in the data [54], a polychoric correlation matrix and the Weighted Least-Squares Means and Variance Adjusted (WLSMV) estimation method were chosen. This approach aligns with previous psychometric studies conducted on Australian [10], Turkish [46] and Chilean [47] samples, which determined a second-order structure with five correlated factors. In the present study, it was proposed to estimate a CFA- bifactor with the 38 items of the SCASIM-St. This model comprises a general factor representing school climate and five specific factors.

A bifactor model is particularly useful for identifying the influence of a general factor versus specific factors, allowing for a more comprehensive view of the underlying structure of a scale. An advantage of this model over a traditional second-order model is that it allows the general factor and the specific factors to directly explain the variability in the items, unlike the second-order model, where the effect of the second-order factor is mediated by the first-order factors; in addition, in bifactor models, it is possible to evaluate the degree of unidimensionality or multidimensionality of the scale [55]. Finally, in bifactor models, it is possible to evaluate the relationship between the set of factors, general and specific, with other external factors, allowing more evidence of criterion validity to be added. In this case, the relationship between these factors and the two dimensions that form the AIA-A will be evaluated.

To evaluate the CFA models, indices such as the WLSMV-χ^2^, the Comparative Fit Index (CFI), the Tucker–Lewis Index (TLI), the Root-Mean-Square Error of Approximation (RMSEA) and the Standardized Root-Mean-Squared Residual (SRMR) were used. Values ≥ 0.96 for the CFI and TLI [56], ≤0.05 for the RMSEA [57] and ≤0.08 for the SRMR [58] were considered reasonable. To assess the essential unidimensionality of the SCASIM-St using the bifactor model, the Explained Common Variance (ECV) and Percentage of Uncontaminated Correlations (PUCs) were used [55,59,60]. ECV values > 0.70 and PUC values > 0.70 suggest a predominantly unidimensional structure [61]. If the PUC exceeds 0.80, the influence of the ECV values on the interpretation of unidimensionality is reduced [62].

In addition, to evaluate the evidence of the reliability of the model, hierarchical omega (ωh) and hierarchical omega-specific (ωhs) reliability coefficients were estimated; these estimators are better than the traditional alpha coefficient for estimating the reliability of the factors of interest in the model because they take into account the multidimensional structure underlying the data [61,62,63,64]. ωh estimates the strength of the general factor in structural equation models [65,66], with values >0.75 indicating the predominance of a general factor [62], and ωhs evaluates the variance explained by each specific dimension controlling for the general factor, where values >0.30 allow us to interpret specific factors’ reliably [67]. Finally, the following indexes were estimated: factor determinancy, which represents the correlation between the factorial scores and the factor, suggesting it can be used to estimate the factorial scores as long as this index is greater than 0.90, and the H index, which measures the replicability of the construct [54] and evaluates the quality with which the set of items represents the latent variable to be measured. For this index, it is recommended that the values be greater than 0.90 [61].

## 3. Results

### 3.1. Descriptive Analysis

Table 1 shows the main descriptive statistics of the SCASIM-St items. The items with the highest means were items 11 (M = 4.34; SD = 0.714) and 22 (M = 4.32; SD = 0.743). On the other hand, the items with the lowest means included items 35 (M = 3.47; SD = 1.043) and 36 (M = 3.49; SD = 1.04), suggesting a trend toward lower responses. Along the same lines, the items with the highest skewness were items 22 (skewness = −1.371) and 11 (skewness = −1.293). Similarly the highest values for kurtosis were for items 22 (kurtosis = 3.376) and 11 (kurtosis = 3.131), suggesting a more extreme concentration of responses compared to a normal distribution. Regarding the evaluation of the fit to a normal distribution, the Kolmogorov–Smirnov test with Lilifors correction presents a *p* < 0.001 for all the items, leading to a rejection of the hypothesis of normality in the distribution of responses for each item. This suggests that the application of estimation methods based on assumptions of normality may not be adequate.

### 3.2. Evaluation of the Factor Structure

When evaluating the dimensional structure of the SCASIM-St using the original second-order model, WLSMV-χ^2^ = 3765.976 (df = 660) with *p* < 0.001 was obtained. The CFI and TLI indices, with values of 0.972 and 0.970, respectively, indicate a good fit of the model since they are close to the ideal threshold of one. The RMSEA shows a value of 0.050 (C.I. 90% = 0.049–0.052), placing it at the upper limit of the “good fit” range, which is generally considered up to 0.05. Likewise, the SRMR, with a value of 0.031, also supports the good fit of the model.

For the bifactor model, the fit indexes show an improvement compared to the original model. The WLSMV-χ^2^ decreases to 3210.617 (df = 627) with *p* < 0.001, and the CFI and TLI indices increase slightly to 0.977 and 0.974, respectively, suggesting an even more robust fit. The RMSEA drops to 0.047 (C.I. 90% = 0.046–0.049), reinforcing the evidence of a good fit. The SRMR also shows an improvement with a value of 0.027.

These results show that both models have an adequate ability to reproduce the observed data, although the bifactor model shows a slight superiority in all the indices evaluated. This suggests that the bifactor structure may be a more accurate representation of the SCASIM-St data, providing a slightly better fit compared to the original second-order model.

Table 2 shows the factor saturations of the two models evaluated. In the bifactor model, the general factor of school climate (SC) stands out, with saturations ranging from a minimum of 0.538 for item 7 to a maximum of 0.79 for items 15 and 16. As for the specific factors, SSRs and SI stand out in particular, showing comparatively higher saturations than the other specific factors. For SSRs, the highest saturations are observed in items 2 (0.599) and 6 (0.596), while the lowest are in items 7 (0.482). In the case of SI, items 36 (0.648) and 35 (0.645) present the highest saturations, while item 38 presents the lowest value (0.264). When evaluating the saturations in the second-order model, the values with the highest discriminative capacity correspond to items 34 (0.927) and 36 (0.904) for the SI factor, which is not part of the first-order factors; similarly, the other first-order factors that are part of the second-order model present relatively high but slightly lower saturations. When evaluating the second-order saturations, values between 0.713 for SSRs and 0.934 for SVAs are observed, indicating a strong relationship between the second-order construct and the first-order factors. The correlation between the second-order factor and the SI factor is 0.763, indicating a positive and high-magnitude relationship.

### 3.3. Reliability Evidence, ECV and PUC

Regarding the quality of the General Factor to explain the common variance of the model, an ECV of 0.703 was observed, indicating that approximately 70% of the common variance of the model can be attributed to the general school climate factor, leaving 30% to the specific factors. Meanwhile, the PUC value was 0.817. These results indicate the existence of a strong general factor with a certain degree of multidimensionality.

In relation to the reliability estimates for the instrument scores, a hierarchical omega value of 0.890, a determinacy factor value of 0.964 and an H-index of 0.974 were observed. Meanwhile, for the specific factors, the reliability by subscale omega for SSRs was 0.435; for SStaffRs, it was 0.207; for AE, it was 0.243; for SVAs, it was 0.104; and finally, for SI, it was 0.362. These results show adequate reliability for the general factor but are not entirely satisfactory for the specific factors.

### 3.4. Evidence of Criterion Validity

When evaluating the external criterion validity of the SCASIM-St, using a model that includes both the bifactor version and an external construct, specifically the AAI-A scale, a satisfactory overall fit was found. The model fit indices are chi-square = 4211.181 (df = 983), *p* < 0.001; CFI = 0.976; TLI = 0.973; RMSEA = 0.042 (C.I. 90% = 0.041–0.044); and SRMR = 0.031, indicating the adequacy of the proposed model for the data.

Regarding correlations, a strong positive relationship was identified between the general school climate factor and positive attitude toward authority, with a correlation of r = 0.758 with *p* < 0.001. On the other hand, a negative relationship, although of a relatively low magnitude, was observed with the positive attitude toward transgression, showing an r = −0.196 with *p* < 0.001. Regarding the specific factors, statistically significant relationships were only found between SStaffR and positive attitude to transgression with a correlation of r = −0.103 at *p* = 0.017 and between SI and positive attitude to transgression with an r = −0.059 at *p* = 0.047. In addition, a negative correlation was observed between positive attitude to authority and positive attitude to transgression, with an r = −0.266 at *p* < 0.001. All these correlations are aligned with the hypotheses posed based on the literature review.

This analysis of the correlations between the factors of the different instruments provides robust evidence of the criterion validity for the model examined, demonstrating that the relationships between the constructs are not only statistically significant but also follow the theoretically expected directions.

## 4. Discussion

The objective of this study was to analyze the psychometric properties of the SCASIM-St from a bifactor approach, evaluating the general factor influence of school climate versus five specific factors of the original theoretical proposal [10]. The results fully demonstrate the achievement of this objective by presenting evidence of validity and reliability for a sample of Chilean students.

In relation to the first hypothesis (H1), the goodness-of-fit indices showed satisfactory results, providing evidence that the factorial structure of the 38 items is consistent with the second-order theoretical proposal and, therefore, is plausible for the sample of Chilean adolescents. These results are considered consistent with SCASIM-St applications conducted in countries such as Australia, Turkey and Chile [10,46,47].

In relation to the second hypothesis (H2), which stated that the SCASIM-St scores would present a bifactor structure with a predominance of a general school climate factor and five specific factors, the results showed goodness-of-fit indices with slightly higher values with regard to the second-order model proposed in H1, confirming the bifactor structure with a strong general factor and five specific factors. The results showed goodness-of-fit indices with slightly higher values with respect to the second-order model proposed in H1, ratifying the bifactor structure with a strong general factor. The high value of the ECV statistic supports the idea that the structure is essentially unidimensional with some degree of multidimensionality. In relation to the reliability evidence (H3), the results show, by means of the hierarchical omega estimation, the adequate reliability of the instrument scores to measure the general factor, results that are in line with the values of the determinacy factor and the H-index. In contrast, the reliability estimates for the specific factors are not entirely satisfactory since only the factors of Student–Student Relations (SSRs) and School identification (SI) presented values of ωhs greater than 0.30.

Concerning the fourth hypothesis (H4), which contrasted the evidence of external criterion validity, evaluating the degree of relationship between the general factor of the SCASIM-St with the two factors of the AIA-A attitude toward authority scale. The result of this analysis showed that the general school climate factor presented negative and statistically significant correlations with the positive attitude toward the transgression factor. On the other hand, the general factor score presented positive correlations of high magnitude and statistical significance with the positive attitude toward the authority factor. These findings coincide with research indicating that those students who show high scores in transgression toward norms present problems in interacting and establishing adequate relationships with peers and other members of the school community [30]. In addition, they display violence toward their parents [31]. In contrast, a positive attitude toward norms favors academic success [42], psychosocial adjustment [32] and a good school climate [68].

The results of this study do not directly contradict the theoretical approaches of the SCASIM-St or previous validation studies of its scores; this is because H1 was satisfactorily fulfilled. Instead, the findings provide a new interpretation that is compatible with the data, in line with H2. This interpretation allows the values of the scale to be considered globally and is a good measure of the general perception that students have of the school climate construct. At the same time, it respects the existence of the multidimensionality of the construct represented by the specific factors, some of which, such as the relationship between students and school identification, have a greater capacity to be interpretable independently of the general school climate factor. As shown in the evidence of external criterion validity, a great advantage of this modelling is its ability to simultaneously assess the relationships of all SCASIM-St factors with other external factors, not limiting itself exclusively to the general factor, as is the case when working with second-order modelling [55].

### 4.1. Contributions for Practice

In addition, the findings of this research contribute to school institutions and public policies, as they contribute to decision-making processes and allow for evaluation and consequent intervention to improve school climate [14,22,69]. This allows multidisciplinary teams to work with the educational community to achieve social, emotional and academic benefits. In this sense, a positive school climate enables students to adapt and develop adequately while, at the same time, teachers perform their work better [12,13,17,31].

### 4.2. Limitations and Future Research

With respect to the limitations of this study, it should be noted that the data were obtained using a cross-sectional design. In this type of design, data collection is performed at a single point in time and lacks longitudinal follow-up, which hinders obtaining more solid evidence about the validity of the results.

Future lines of research should include the application of longitudinal research designs to strengthen the evidence of the validity and reliability of the scale. In addition, new studies should be applied with larger samples that consider diverse international populations. Along the same lines, future work can deepen multilevel analysis by evaluating the bifactor structure of the SCASIM-St and providing new evidence of its validity. It becomes interesting, once sufficient evidence is consolidated, to continue deepening it; therefore, it would be relevant to carry out an evaluation of the invariance between different groups of interest, particularly if a comparison between these groups in terms of the scores of the instrument is planned. Finally, it would be beneficial to carry out future Chilean studies that add more evidence of criterion validity, for example, by evaluating the relationship between school climate and other relevant constructs, such as psychological well-being, mental health, school engagement and academic success.

## 5. Conclusions

This study provided valuable evidence regarding a new interpretation of the factor structure of the SCASIM-St. Currently, most interpretations of the scale scores are based on a multidimensional structure; in contrast, this study proposes a unidimensional structure, an aspect that will facilitate the understanding of school climate scores, simplify the estimation of scores for the professional environment and introduce a unidimensional factor structure of the construct, providing a parsimonious interpretation of school climate for the academic environment.

In relation to its contributions to educational practice, this study offers a new interpretation of the scores, providing a simplification of the estimation of the SCASIM-St scores for professionals in the area, allowing the 38 items to be summed, thus obtaining a parsimonious index of school climate. This approach can be instrumental in improving communication and the overall understanding of school climate in an educational setting. To maximize the impact of these findings on educational practice, it is suggested that educational professionals consider adopting this new interpretation in their school climate assessments.

## Figures and Tables

**Table 1:** Descriptive statistics of SCASIM-St items.

Items	M	SD	Skewness	Kurtosis
Item1	3.65	0.880	−0.506	0.421
Item2	3.82	0.815	−0.679	0.982
Item3	3.73	0.821	−0.522	0.524
Item4	3.76	0.795	−0.481	0.500
Item5	3.61	0.798	−0.280	0.200
Item6	3.69	0.807	−0.491	0.624
Item7	3.86	0.870	−0.669	0.526
Item8	4.17	0.769	−1.015	1.991
Item9	4.20	0.737	−0.883	1.474
Item10	4.16	0.770	−0.931	1.552
Item11	4.34	0.714	−1.293	3.131
Item12	3.98	0.840	−0.839	1.100
Item13	3.79	0.897	−0.653	0.554
Item14	4.06	0.802	−0.867	1.345
Item15	4.05	0.812	−0.895	1.335
Item16	3.98	0.854	−0.792	0.846
Item17	4.02	0.874	−0.924	1.064
Item18	4.10	0.807	−0.959	1.440
Item19	4.07	0.837	−0.943	1.262
Item20	4.00	0.775	−0.764	1.250
Item21	4.16	0.749	−0.96	1.922
Item22	4.32	0.743	−1.371	3.376
Item23	4.19	0.858	−1.094	1.327
Item24	4.06	0.8230	−0.797	0.752
Item25	3.71	0.867	−0.456	0.303
Item26	3.59	0.935	−0.487	0.169
Item27	3.72	0.919	−0.554	0.253
Item28	3.73	0.875	−0.511	0.209
Item29	3.83	0.821	−0.546	0.637
Item30	3.66	0.949	−0.557	0.248
Item31	4.07	0.776	−0.943	1.728
Item32	3.97	0.819	−0.829	1.193
Item33	3.79	0.998	−0.708	0.258
Item34	3.87	0.949	−0.789	0.618
Item35	3.47	1.043	−0.338	−0.241
Item36	3.49	1.040	−0.398	−0.165
Item37	3.69	1.010	−0.652	0.225
Item38	4.20	0.791	−1.139	2.254

Note: M, mean; SD, standard deviation. Standard error of skewness = 0.057. Standard error of kurtosis = 0.113. The *p*-value of the Kolmogorov–Smirnov test with Lilifors correction was <0.001 for all items.

**Table 2:** Saturation CFA bifactor model (second-order model saturations in parentheses).

Ítem	SC	SSR	SStaffR	AE	SVA	SI
1	0.586	0.53 (0.807)				
2	0.557	0.599 (0.799)				
3	0.573	0.542 (0.797)				
4	0.547	0.58 (0.782)				
5	0.594	0.587 (0.835)				
6	0.592	0.596 (0.835)				
7	0.538	0.482 (0.741)				
8	0.763		0.361 (0.855)			
9	0.709		0.453 (0.824)			
10	0.763		0.427 (0.871)			
11	0.735		0.469 (0.855)			
12	0.729		0.421 (0.835)			
13	0.667		0.29 (0.742)			
14	0.783		0.395 (0.883)			
15	0.79		0.433 (0.898)			
16	0.79		0.385 (0.887)			
17	0.73			0.392 (0.843)		
18	0.716			0.447 (0.839)		
19	0.667			0.389 (0.776)		
20	0.692			0.369 (0.799)		
21	0.587			0.486 (0.713)		
22	0.714			0.529 (0.856)		
23	0.733			0.416 (0.851)		
24	0.720			0.349 (0.824)		
25	0.741				0.105 (0.773)	
26	0.776				0.196 (0.818)	
27	0.779				0.245 (0.827)	
28	0.742				0.36 (0.802)	
29	0.749				0.176 (0.788)	
30	0.581				0.26 (0.626)	
31	0.759				0.387 (0.826)	
32	0.721				0.424 (0.791)	
33	0.707					0.540 (0.901)
34	0.724					0.563 (0.927)
35	0.642					0.645 (0.887)
36	0.657					0.648 (0.904)
37	0.658					0.601 (0.881)
38	0.621					0.264 (0.743)
Saturations in the second order factor						
		SSR	SStaffR	AE	SVA	
		0.713	0.881	0.856	0.934	
Correlation between the second order factor and SI = 0.763						

Note: SC = school climate; SSRs = Student–Student Relations; SStaffRs = Student–Staff Relations; AE = Academic Emphasis; SVAs = Shared Values and Approach; SI = School Identification.

## Data Availability

The dataset for the study is available from the corresponding author upon reasonable request due to ethical restrictions.

## Supplementary Materials

The following supporting information can be downloaded at: https://www.mdpi.com/article/10.3390/children11010087/s1, Table S1: Items in the original English version of SCASIM-ST and their adapted version in Spanish. Table S2: Estimates under the bifactor model of factor loadings. Standard Errors. Z and *p* value. Table S3: Estimates under the second-order model of factor loadings. Standard Errors. Z and *p* value.
