# Supplementary material for: Psychometric Evaluation of the School Climate and School Identification Measure—Student on Chilean Students: A Bifactor Model Approach
Source: Children (Basel). 2024 Jan 11;11(1):87. doi: 10.3390/children11010087 (PMC10813966; doi:10.3390/children11010087)
Supplement: Supplementary file 1 [file children-11-00087-s001.zip › children-2795774-supplementary.pdf]

Table S1. Items in the original English version of SCASIM-ST and their adapted version in Spanish.

Student-student relationships / Relaciones estudiante-estudiante
it1 Students care about each other / Los estudiantes se cuidan unos a otros
it2 Students are friendly to each other / Los estudiantes son amigables entre si
it3 Students go out of their way to help each other / Los estudiantes buscan la forma de ayudarse unos a otros
it4 Students treat each other with respect / Los estudiantes se tratan con respeto entre si
it5 Students are fair to each other / Los estudiantes son justos entre sí
it6 Students show understanding to each other / Los estudiantes muestran comprensión entre ellos
it7 Students are accepting of each others' differences / Los estudiantes aceptan diferencias de los demás
Student-staff relationships / Relaciones estudiantes-personal
it8 Staff care about students / El personal cuida a los estudiantes
it9 Staff are friendly to students / El personal es amigable con los estudiantes
it10 Staff go out of their way to help students / El personal busca formas de ayudar a los estudiantes
it11 Staff treat students with respect / El personal trata a los estudiantes con respeto
it12 Staff listen to what students have to say most of the time / El personal escucha lo que los estudiantes tienen que decir la mayoría del tiempo
it13 Staff involve students in decisions and planning / El personal involucra a los estudiantes en las decisiones y planificación
it14 Staff are fair in their dealing with students / El personal es justo en su trato con los estudiantes
it15 Staff show understanding to students / El personal muestra comprensión a los estudiantes
it16 Staff take students' concerns seriously / El personal toma en serio las preocupaciones de los estudiantes
Academic emphasis / Énfasis académico
it17 Teachers encourage students to try out new ideas (think independently) / Los profesores animan a los estudiantes a probar nuevas ideas Pensar independientemente)
it18 Teachers challenge students to do better / Los profesores desafían a los estudiantes a hacerlo mejor
it19 Teachers are willing to give students extra help on school work if needed / Los profesores están dispuestos a dar una ayuda extra en el trabajo escolar si es necesario
it20 Teachers set high standards for learning in their classes / Los profesores establecen altos estándares de aprendizaje en sus clases
it21 Teachers expect everyone to work hard / Los profesores esperan que todos trabajen mucho
it22 Teachers want every student to do their best / Los profesores quieren que cada estudiante haga su mejor esfuerzo
it23 Teachers believe that every student can be a success / Los profesores creen que cada estudiante puede ser un éxito
it24 Teachers give useful feedback / Los profesores dan una retroalimentación útil
Shared values and approach / Valores y enfoques compartidos
it25 Students and staff are working towards the same goals/ Los estudiantes y el personal luchan por los mismos objetivos
it26 There is a sense that we are all on the same team / Hay un sentido de pertenencia y que todos estamos en el mismo equipo
it27 There is school spirit and pride / Hay espíritu y orgullo escolar
it28 The school values and goals are well understood / Los valores y objetivos de la escuela son bien entendidos
it29 New students and staff are made to feel welcome as part of the group / Los nuevos estudiantes y personal son hechos sentir bienvenidos como parte del grupo
it30 Student and staff who uphold the values of the school are recognized and celebrated / El estudiante y personal que defiende los valores de la escuela son reconocidos y celebrados
it31 The expectations and rules are clear / Las expectativas y reglas son claras
it32 The rules related to discipline are clear and well-understood by staff and students / Las reglas relacionadas con la disciplina son claras y bien entendidas por el personal y los estudiantes
School identification / Identificación escolar

it33 Being a part of this school is important to me / Ser parte de esta escuela es importante para mi
it34 I am happy to be a part of this school / Soy feliz de ser parte de esta escuela
it35 I feel a strong connection with this school / Siento una fuerte conexión con esta escuela
it36 I identify with this school / Me identifico con esta escuela
it37 I feel I belong at this school / Siento que pertenezco a esta escuela
it38 I care about this school / Cuido a esta escuela

Response scale 1 = strongly disagree; 2 = in disagreement; 3 = neither agree nor disagree; 4 = agree; 5 = totally agree

Escala de respuesta 1 = totalmente en desacuerdo; 2 = en desacuerdo; 3 = ni de acuerdo. ni en desacuerdo; 4 = de acuerdo; 5 = totalmente de acuerdo

Table S2. Estimates under the bifactor model of factor loadings. Standard Errors. Z and p value.

Factor	Estimate	Standard Error	Est./S.E	p value
SSR				
Item1	0.530	0.016	32.645	<0.001
Item2	0.599	0.015	38.669	<0.001
Item3	0.542	0.016	33.549	<0.001
Item4	0.580	0.015	38.446	<0.001
Item5	0.587	0.015	38.760	<0.001
Item6	0.596	0.015	40.323	<0.001
Item7	0.482	0.018	27.169	<0.001
SStaffR				
Item8	0.361	0.020	18.369	<0.001
Item9	0.453	0.019	23.457	<0.001
Item10	0.427	0.018	23.823	<0.001
Item11	0.469	0.018	25.410	<0.001
Item12	0.421	0.018	23.685	<0.001
Item13	0.290	0.021	13.898	<0.001
Item14	0.395	0.017	22.550	<0.001
Item15	0.433	0.017	25.455	<0.001
Item16	0.385	0.017	22.160	<0.001
AE				
Item17	0.392	0.018	21.274	<0.001
Item18	0.447	0.018	24.430	<0.001
Item19	0.389	0.020	19.117	<0.001
Item20	0.369	0.020	18.314	<0.001
Item21	0.486	0.019	25.254	<0.001
Item22	0.529	0.018	29.183	<0.001
Item23	0.416	0.017	24.352	<0.001
Item24	0.349	0.020	17.429	<0.001
SVA				
Item25	0.105	0.023	4.493	<0.001
Item26	0.196	0.022	8.971	<0.001
Item27	0.245	0.022	11.235	<0.001
Item28	0.360	0.021	16.796	<0.001
Item29	0.176	0.024	7.432	<0.001
Item30	0.260	0.025	10.554	<0.001
Item31	0.387	0.021	18.128	<0.001
Item32	0.424	0.022	19.106	<0.001
SI				
Item33	0.540	0.015	36.968	<0.001
Item34	0.563	0.014	39.769	<0.001
Item35	0.645	0.014	47.333	<0.001
Item36	0.648	0.013	48.271	<0.001
Item37	0.601	0.014	44.214	<0.001
Item38	0.264	0.021	12.709	<0.001
SC				
Item1	0.586	0.017	35.430	<0.001
Item2	0.557	0.018	31.492	<0.001
Item3	0.573	0.017	33.619	<0.001
Item4	0.547	0.018	31.259	<0.001
Item5	0.594	0.016	37.204	<0.001
Item6	0.592	0.017	35.655	<0.001
Item7	0.538	0.018	29.846	<0.001

Item8	0.763	0.013	59.396	<0.001
Item9	0.709	0.015	48.053	<0.001
Item10	0.763	0.012	61.092	<0.001
Item11	0.735	0.014	51.429	<0.001
Item12	0.729	0.013	56.077	<0.001
Item13	0.667	0.015	45.928	<0.001
Item14	0.783	0.011	69.810	<0.001
Item15	0.790	0.011	72.359	<0.001
Item16	0.790	0.011	71.535	<0.001
Item17	0.730	0.013	57.858	<0.001
Item18	0.716	0.013	53.957	<0.001
Item19	0.667	0.015	45.852	<0.001
Item20	0.692	0.014	47.972	<0.001
Item21	0.587	0.017	34.488	<0.001
Item22	0.714	0.014	49.586	<0.001
Item23	0.733	0.013	56.622	<0.001
Item24	0.720	0.013	55.246	<0.001
Item25	0.741	0.013	57.130	<0.001
Item26	0.776	0.011	71.006	<0.001
Item27	0.779	0.011	70.378	<0.001
Item28	0.742	0.012	60.282	<0.001
Item29	0.749	0.012	60.427	<0.001
Item30	0.581	0.017	35.044	<0.001
Item31	0.759	0.012	63.154	<0.001
Item32	0.721	0.013	54.526	<0.001
Item33	0.707	0.013	53.816	<0.001
Item34	0.724	0.013	55.968	<0.001
Item35	0.642	0.015	42.773	<0.001
Item36	0.657	0.014	46.062	<0.001
Item37	0.658	0.014	46.603	<0.001
Item38	0.621	0.017	37.152	<0.001

Table S3. Estimates under the second-order model of factor loadings. Standard Errors. Z and p value.

Factor	Estimate	Standard Error	Est./S.E	p value
SSR				
Item1	0.807	0.012	67.028	<0.001
Item2	0.799	0.012	68.349	<0.001
Item3	0.797	0.012	66.187	<0.001
Item4	0.782	0.012	63.613	<0.001
Item5	0.835	0.011	78.921	<0.001
Item6	0.835	0.011	77.760	<0.001
Item7	0.741	0.014	53.364	<0.001
SStaffR				
Item8	0.855	0.009	98.222	<0.001
Item9	0.824	0.010	82.033	<0.001
Item10	0.871	0.008	115.366	<0.001
Item11	0.855	0.009	91.982	<0.001
Item12	0.835	0.009	96.632	<0.001
Item13	0.742	0.012	61.616	<0.001
Item14	0.883	0.007	128.135	<0.001
Item15	0.898	0.006	148.533	<0.001
Item16	0.887	0.007	135.586	<0.001
AE				
Item17	0.843	0.009	93.483	<0.001
Item18	0.839	0.009	91.021	<0.001
Item19	0.776	0.011	68.101	<0.001
Item20	0.799	0.011	69.533	<0.001
Item21	0.713	0.014	51.758	<0.001
Item22	0.856	0.009	94.240	<0.001
Item23	0.851	0.009	90.244	<0.001
Item24	0.824	0.010	81.608	<0.001
SVA				
Item25	0.773	0.012	64.326	<0.001
Item26	0.818	0.009	87.199	<0.001
Item27	0.827	0.009	90.807	<0.001
Item28	0.802	0.010	83.220	<0.001
Item29	0.788	0.011	72.699	<0.001
Item30	0.626	0.015	40.927	<0.001
Item31	0.826	0.009	88.556	<0.001
Item32	0.791	0.010	76.475	<0.001
SI				
Item33	0.901	0.006	148.376	<0.001
Item34	0.927	0.006	161.667	<0.001
Item35	0.887	0.006	140.827	<0.001
Item36	0.904	0.005	166.575	<0.001
Item37	0.881	0.006	136.269	<0.001
Item38	0.743	0.015	48.423	<0.001
SC				
SSRR	0.713	0.014	52.153	<0.001
SStaffR	0.881	0.008	108.587	<0.001
AE	0.856	0.009	92.466	<0.001
SVA	0.934	0.006	158.673	<0.001
Correlation SC with SI	Estimate	Standard Error	Est./S.E	p value
SC				
SI	0.763	0.011	68.504	<0.001
